# Chemistry and Physiology

**Published:** 1859-05

**Authors:** 


CHIEFLY FRENCH, GERMAN, AND ITALIAN; OR, BI-MONTHLY NOVELTIES IN MEDICAL, SURGICAL, OBSTETRICAL, AND CHEMICAL SCIENCE. BY EMIL FISCHER, M.D.

### CHEMISTRY AND PHYSIOLOGY.

### I.—*On the Locality in which the Impressions of Taste are received.* By DRS. KLAATSCH and STICH.

Drs. Klaatsch and Stich have, after many experiments, arrived at the conclusion that the locality which receives the gustatory impressions is a small space running around the tongue near its margin; in some persons it is hardly two lines wide, in others, it is double this width; in most cases it occupies just the middle of the margin of the tongue; in some, it approaches nearer to its upper surface; only in very few instances, it lies closer to the under surface. In investigating the surface of the tongue, care must be taken that the substances applied to it do not come too near its margin; it is therefore best to commence by brushing them on the middle of the tongue, and to approach the margin gradually. This experiment will show that, in most individuals, the application can be continued until it reaches the margin of the tongue, without taste becoming perceptible, while in others, the perception of taste commences at the distance of a line from the margin. Gustatory impressions are also perceived through the medium of the soft palate. That the pharynx and tonsils do not possess the sense of taste, is proved by the fact that in touching the throat with nitrate of silver, the patients do not experience the disagreeable taste of this substance, provided that they keep their tongue at rest, and that this organ, as well as the soft palate, are avoided in the application.—(*Virchow's Archiv. f. Patholog. Anatomie, Physiologie und Klinische Medicin*, xiv. 3, 4.)

II.—*Physiological Researches on Digestion.* By Dr. Busch.

The author had an opportunity of studying the physiological phenomena of digestion, in a woman suffering from an intestinal fistula which communicated with the upper part of the jejunum, and had been produced by a penetrating wound of the abdomen. The patient had a voracious appetite and devoured an enormous quantity of food, the greater part of which escaped through the orifice of the fistula, mixed with bile and the gastric, intestinal, and pancreatic juices; the orifice of the lower portion of the intestines, being at a great distance from the upper, did not receive an atom of aliment. This state of affairs had produced extreme emaciation and debility, accompanied by a great tendency to sleep, hoarseness, etc.

Dr. Busch concluded to inject aliment into the lower portion of the intestines; he used especially concentrated broths, to which beaten eggs, and sometimes hard eggs and meat were added. This treatment revived the strength of the patient and caused her to gain more flesh, a curious result, which shows that the absorption and assimilation of food is, at least for a time, possible without the aid of the gastric, duodenal and pancreatic secretions, and of bile. Just as remarkable is the fact, that the ingestion of food through the mouth alone, was sufficient to keep this woman alive, after she had once regained her strength by the mode of alimentation employed by Dr. Busch.

The results of the experiments made by the author on the patient may be resumed in the following propositions:—1. The sensation of hunger is ordinarily composed of two distinct impressions: the one has its origin in the general nervous system, which feels, so to say, the necessity of being renewed; the other is exclusively produced by the nerves of the digestive organs. 2. The peristaltic movements of the intestines are intermittent, without there being any regularity recognizable in the alternation of repose and contraction. Dr. Busch could not exactly determine the energy of these movements, but has ascertained that it is superior to the pressure of a column of water two feet high; he also has seen that the lower end of the intestines often presented very manifest anti-peristaltic movements. 3. The intestinal juice is never produced but in very small quantity; its reaction is always alkaline; it contains from 3·87 to 7·4 per cent. of solid matter. 4. Its influence upon the digestion of protein substances is not to be doubted, but it is always accompanied by the putrefaction of these substances. 5. The intestinal juice transforms starch into grape sugar. 6. It does not transform cane sugar into grape sugar. 7. Grape sugar is absorbed without being modified, and does not pass into the urine. 8. The absorption of fatty matter is impossible, or at least, very incomplete, if it is not subjected to the action of the gastric and pancreatic juices. 9. The reaction of the mixture of secretions which escaped from the upper end, in state of fasting, was nearly always neutral, rarely a little acid or alkaline. 10. This mixture never shows the reaction of the saliva; we may therefore conclude that the saliva is absorbed before arriving at the jejunum. 11. The same mixture contains 2·48 per cent. of solid substances. 12. The first portions of food introduced into the stomach show themselves in the jejunum at the end of fifteen to thirty minutes. 13. The solutions of cane sugar disappear for the greater part in the uppermost portion of the digestive tube; when the cane sugar arrives in the jejunum it is transformed into grape sugar. 14. White of eggs, in a raw state, is also absorbed in great part by the stomach and the contiguous part of the intestines; on arrival in the jejunum it does not undergo transformation. 15. Gum traverses the small intestines without being modified. 16. Gelatine is dissolved and does not coagulate consecutively; it is absorbed in great part. 17. A part of the casein contained in milk arrives in the jejunum without being coagulated. 18. The mixture of digestive liquids contained in the duodenum emulsifies perfectly the fatty matter, if its reaction is alkaline; if it is acid, the emulsion is incomplete. 19. This mixture has the property of digesting protein substances. 20. The quantity of digestive liquids which arrives as far as the jejunum in twenty-four hours, is equal at least to the twenty-seventh part of the weight of the body.—(*Archiv. für Pathologische Anatomie*, xiv., 1858, and *Archives Générales*, February, 1859.)

III.—*On a New Function of the Placenta and of the Amnios.* By M. CLAUDE BERNARD. (Communication made to the *Académie des Sciences*, January 10th, 1859.)

M. Claude Bernard had pointed out, a long time ago, the existence of glucose in the liquor amnii. In pursuing his researches on the origin of this substance, he found that the placenta of certain mammalia contains, in the normal state, a considerable quantity of the sugar-forming substance which he has discovered to be constantly present in the liver. The ruminantia seemed to offer an exception; but M. Claude Bernard has finally overcome this last difficulty. In these animals the sugar-forming substance is produced in certain points of the amnios, instead of being found in the placenta.

The sugar-forming matter of fœtal life, whatever be the species under observation, is produced by cells which, in their histological characters, are similar to the cells of the liver in full-grown animals.

In the rodentia, these cells are found between the fœtal and the maternal placenta. In the ruminantia, whitish spots are seen on the amnios, which have either been left unnoticed by the observers, or have been mistaken for pathological productions of epithelial nature. These white spots are constituted by agglomerations of cells similar to the preceding.

On examining these spots with the microscope, they are found to be composed of a kind of papillæ filled with cells and circumscribed by an amorphous membrane. Iodine imparts to the cellular contents a rose color.

The layer of cells separating the fœtal and maternal placentas in the rodentia, and the papillæ containing the cells in the ruminantia, are developed from the earliest period of fœtal life, and begin their function immediately. Resembling the hepatic cells in their form, they perform a similar office, at least as far as the production of glucose is concerned, for M. Bernard has not yet terminated his researches with regard to the possible secretion of bile by these organs. Thus it is a true liver, the existence of which was absolutely unknown until the present time. While these organs are active, the internal liver of the fœtus is in a rudimentary state; its cells, which are quite embryonic, produce neither sugar-forming substance nor glucose. Later, they change their aspect, assume their normal form and dimensions, and commence to secrete sugar-forming matter. At the same time the hepatic cells of the placenta and of the amnios become atrophied, and disappear afterwards.

The glucogenous matter exists thus in animals from the beginning of fœtal life, and seems, consequently, to be a necessary element of the first cellular formations. In the same manner, the secretion of this matter continues during the whole life for the sake of the renovation of the histological elements of the grown-up animal; for the acts of nutrition cannot, in a physiological point of view, differ essentially from those of the first development.—(*Gazette Hebdomad.*, January 14th, 1859.)

IV.—*Observations on the Changes of the Urine in Disease.* By Dr. Brattler.

Dr. Brattler has made a series of very accurate investigations on the changes of the urine in typhus, morbilli, scarlatina, diseases of the heart, etc., which he laid down in an elaborate treatise, entitled "*Beitrag zur Urologie im Kranken Zustande;*" München, 1858, *Joh. Palm's Hofbuchhandlung.*

The author gives the following summary of his urological observations :—

Casting a retrospective glance upon our investigations and experiments, we find that the urine does not suffer in disease any changes peculiar to the different morbid conditions, but that these changes are in relation with definite processes going on in the organism. The urine of a case of typhus, pneumonia, cholera, or Bright's disease, may have one and the same qualities, for the very reason that certain processes, which modify the secretion of urine, may take place in any of these diseases.

*The quantity of urine.* It is *diminished:* In the commencement of nearly all febrile diseases; in diseases of the kidneys, when the uriniferous tubules are obstructed, (morbus Brightii.)

In diseases in which the organism suffers great losses of serum, as excessive diarrhœa, cholera, copious perspiration.

In diseases of the circulatory and respiratory organs, in consequence of which less blood is furnished to the aortic system, and therefore to the kidneys, as disease of the heart, and pleuritic exudation.

It is *augmented:* By the resorption of hydropic effusions and exudations.

In polydypsia, diabetes insipidus.

*Urea.* It is *diminished:* In the re-convalescence from all acute diseases, in which the organism has suffered a considerable loss of substance through fever, as in this case the nourishment carried into the system is used for the reparation of the lost nitrogenous tissues.

In diseases of the digestive organs which hinder the resorption of the ingesta, as chronic vomiting in atrophy after typhus, and cancer of the stomach.

In diseases of the kidneys, interfering with their functions, (morbus Brightii.)

In diseases of the circulatory and respiratory organs, in consequence of which less blood is furnished to the aortic system, and therefore to the kidneys.

It is *augmented:* In all diseases accompanied by fever, viz., by elevation of temperature. (The frequency of the pulse bears no constant relation to the secretion of urea.) The secretion of urea is the greater the higher the temperature rises.

An exception takes place only when in febrile diseases the function of the kidneys is at the same time interfered with, be it by disease of these organs themselves, or secondarily by the influence of other organs.

In diseases in which the urea has been retained for a long time in the blood by functional disorder of the kidneys, after removal of the difficulty, as morbus Brightii, cholera, and disease of the heart.

By the resorption of hydropic effusions, as morbus Brightii, and dropsy from disease of the heart.

*Chlorides.* They are *diminished:*

In all diseases in which exudations or transudations take place, these effusions being rich in chlorides, as typhus, pneumonia, pleuritis, Bright's disease, cholera, acute rheumatism, etc.

In diseases of the digestive organs which hinder the resorption of the ingesta.

In diseases or functional disorders of the kidneys with diminished urinary secretion, as Bright's disease, and disease of the heart.

They are *augmented:* By the resorption of hydropic effusions.

*Phosphoric acid.* It is *diminished:*

In diseases or functional disorders of the kidneys with diminished urinary secretion, as Bright's disease, and disease of the heart.

In diseases of the digestive organs which hinder the resorption of the ingesta.

It is *augmented:* In acute febrile diseases by the increased metamorphosis of tissues containing phosphorus.

The increase of phosphoric acid is, however, not as constant as that of urea. In diseases in which the phosphoric acid has been retained for a long time in the blood by functional disorder of the kidneys, after removal of the difficulty, as Bright's disease, and cholera.

According to *Bence Jones*, in acute nervous diseases, and in osteomalacia.—(*Medicinische Central Zeitung*, 83, 1858.)

